# Spine surgery and readmission: Risk factors in lumbar corpectomy patients

**DOI:** 10.1016/j.xnsj.2025.100587

**Published:** 2025-01-20

**Authors:** Julius Gerstmeyer, Anna Gorbacheva, August Avantaggio, Clifford Pierre, Emre Yilmaz, Thomas A. Schildhauer, Amir Abdul-Jabbar, Rod J Oskouian, Jens R Chapman

**Affiliations:** a Swedish Neuroscience Institute, Swedish Medical Center, Seattle, Washington, USA. 550 17th Avenue, Suite 500, Seattle, WA 98122, United States; b Seattle Science Foundation, 550 17th Avenue, Suite 600, Seattle, WA 98122, United States; c Department of General and Trauma Surgery, BG University Hospital Bergmannsheil, Ruhr University Bochum, Bürkle-de-la-Camp-Platz 1, 44789 Bochum, Germany

**Keywords:** Readmission rate, Corpectomy, Lumbar corpectomy, Risk factors, Spine surgery, Outcomes, Complications

## Abstract

**Background:**

A corpectomy of the lumbar spine is a widely performed surgical procedure with numerous indications. Previous research predominantly focused on various surgical techniques and their outcomes, lacking a general and comprehensive analysis of factors affecting this procedure. With this study, we aimed to assess the all-cause 90-day readmission rate and identify risk factors for adverse events following a lumbar corpectomy.

**Methods:**

Utilizing the 2020 Nationwide Readmissions Database adults (>18 years) were selected by ICD-10 procedure category codes for lumbar corpectomy. Patients with adult deformity or degenerative conditions were excluded due to coding inconsistencies. Demographic information and clinical data, including comorbidities, was extracted. Patients were categorized by their readmission status. The primary outcome was readmission, with multivariable logistic regression analysis used to identify independent risk factors.

**Results:**

A total of 3,238 patients were included, with 20.8% readmitted. The readmission group was significantly older and had higher comorbidity burdens. Malignancy had the greatest odds of readmission (OR 3.172, p=.002), with spondylodiscitis also showing significant association (OR 2.177, p=.030). Fractures were significantly more frequent in the single admission group and not associated with readmission (OR 1.235, p=.551). Medical comorbidities differed significantly between the groups with a variety of them being identified as risk factors.

**Conclusions:**

We established an all-cause 90-day readmission rate of 20.8%, which is in range of other procedures in spine surgery but underscores the severity of lumbar corpectomy. Underlying pathologies have a greater impact on the readmission rate compared to medical comorbidities. These findings highlight the importance of preoperative patient selection, especially when performing more invasive procedures. However, the study's limitations may limit the generalizability of the findings.

## Introduction

A lumbar corpectomy is a relatively major surgical procedure typically performed for serious conditions like malignant formations, infection, trauma, or adult deformity, with recent advancements aiming to reduce invasiveness, complications, and recovery times compared to traditional open approaches [[1], [2], [3], [4]].

While readmission rates and risk factors for other spine pathologies have been studied in the general context of corpectomies, literature specific to a corpectomy of any region of the spine is scarce. Current research predominantly focuses on surgical techniques and their outcomes, highlighting complication rates as high as 32.8% with a surgical revision rate of 5% [[5], [6], [7], [8], [9]]. However, these studies lack a comprehensive analysis of spinal pathologies and comorbidities in conjunction with a corpectomy, especially in the lumbar spine.

Ninety-day readmission rates in spine surgery have been reported as high as 35%, varying significantly based on the underlying pathology or procedure [[10], [11], [12], [13], [14]].

Readmission rates and risk factors for lumbar corpectomies have not yet been studied. Real-life data is helpful in establishing realistic quality parameters, optimizing informed clinical decision making and perhaps most importantly guiding patient management. Such data is relevant to both health care and administrative professionals.

We hypothesized that, despite the relative severity of the procedure, lumbar corpectomies incur readmission rates similar to other spinal procedures.

The aim of this study is to assess the 90-day-all-cause readmission rate for patients who underwent a lumbar corpectomy and to establish risk factors for readmission based on presenting pathology and medical comorbidities.

## Methods

### Study design and data source

Using the 2020 Nationwide Readmissions Database (NRD), Healthcare Cost and Utilization Project (HCUP), Agency for Healthcare Research and Quality, a retrospective cohort analysis was performed. The database captures discharge data from 30 states, accounting for 61.1% of the total United States population and 60.0% of all hospitalizations. Demographic information, admission status, readmission, clinical data, length of stay, in-hospital death as well as comorbidities were extracted. Comorbidities were identified using ICD-10 codes and primary clinical classifications software refined (CCSR) for International Classification of Diseases (ICD)−10 category codes, Version 2023 [15]. To assess the comorbidity burden of each patient, the Elixhauser Comorbidity Index for in-hospital mortality and all-cause 30-day readmission were calculated [16]. Per HCUP agreement, any risk factors, comorbidities or complications were excluded from the analysis, if the incidence were 10 or fewer.

Since the data is publicly available, no approval from an institutional or national research committee was required.

### Population

Adult patients (>18 years) who had undergone a lumbar corpectomy were selected by International Classification of Diseases (ICD)−10-PCS procedures codes, Version 2023.1 [17]. The codes were previously published by Kandregula et al. [18].

We included patients with fractures, malignant formations and spondylodiscitis of the spine. Adult deformity and degenerative conditions were excluded due to inconsistencies and great variety in ICD-10 coding. To reliably calculate a 90-day readmission rate, only patients admitted within the first 9 months of the year were included.

### Study cohorts and outcomes

The study cohorts were categorized into 2 groups based on their readmission status.

Readmissions were identified via *visitlinks*, which can be used for tracking patients across hospitals in a year. Comorbidities present on discharge were identified and selected independently by 2 senior spine surgeons.

### Statistical analysis

All statistical analysis was performed using SPSS (IBM® SPSS Statistics®, Version 29.0.2.0 (20) Armonk, NY, USA). Descriptive analyses are presented as mean with standard deviation (SD), or frequencies with their percentages where appropriate.

To compare categorical variables, the Chi-square test was used. For continuous outcomes, a t-test was used. We also evaluated for high levels of co-linearity. A multivariable logistic analysis was conducted to evaluate the effects of variables on readmission. The effect of each risk factor, controlling for the others in the model, were reported using odds ratios (OR), 95% confidence intervals (CI) and p-value. The level of confidence was set at p=.05.

## Results

In total, 3,238 patients were included in this study, with 675 (20.8%) being readmitted for any cause within 90 days. The average time until readmission was 38.34 (±22.3). Although gender distribution, length of stay and nonelective admission status were similar in both groups, the readmission group was significantly older at 60.5 years (±16.4) compared to the single admission group (54.6 years (±19.1). Notably, the majority of patients were initially admitted nonelectively in both cohorts (Table 1).

**Table 1:** Overview of demographics and payer status.

	Single admission *N*=2563	Readmission <90 days *N*=675	*p*-value
**Demographics**	**N (%) or**	**Mean (± SD)**	
Age	54.6 (±19.1)	60.5 (±16.4)	<.001
Male	1522 (59.4)	380 (56.3)	.147
Length of stay	13.1 (±15.7)	13.1 (±10.1)	.881
Nonelective admission	1927 (75.2)	520 (77)	.319
Time until readmission		38.34 (±22.3)	
Elixhauser in-hospital mortality index	−0.38 (±8.8)	1.97 (±8.3)	<.001
Elixhauser 30 days readmission index	3.61 (±4.8)	5.1 (±5.3)	<.001
**Payer**			
Medicare	962 (37.5)	350 (51.9)	<.001
Medicaid	420 (16.4)	106 (15.7)	.668
Commercial Insurance	859 (33.5)	168 (24.9)	<.001
Self-pay	112 (4.4)	19 (2.8)	.068
No charge	11 (0.4)	*	.285
Other	199 (7.8)	31 (4.6)	.004

⁎ Indicates that value was below HCUP reporting minimum of 11, due to privacy protection guidelines.

Additionally, the Elixhauser indices for both readmission and in-hospital mortality differed significantly between the groups, indicating a greater risk for both events in the readmission group, reflecting a higher comorbidity burden.

Medicare insurance was most frequent in both groups (37.5% single admission; 51.9% readmission), followed by commercial insurance (16.4%; 24.9%). Both were significantly more frequent in the readmission group. No significant differences were observed between Medicaid, self-pay and no charge.

We found several significant differences between the cohorts regarding both spinal pathologies and comorbidities (Table 2). Most corpectomies were performed for patients presenting with fractures. These patients were significantly less frequently readmitted (66.3% single admission vs. 46.1% readmission, p≤.001). Patients treated either for spondylodiscitis or malignancy were significantly more prevalent in the readmission group.

**Table 2:** Spine pathologies and comorbidities.

	Single admission *N*=2563	Readmission <90 days *N*=675	*p*-value
**Spine pathology**	**N (%) or**	**Mean (± SD)**	
Fractures	1699 (66.3)	311(46.1)	<.001
Spondylodiscitis	235 (9.2)	119 (17.7)	<.001
Malignancy	657 (25.6)	260 (38.5)	<.001
**Comorbidities**			
Obesity	439 (17.2)	39 (5.8)	<.001
Diabetes mellitus type 2	507 (19.8)	141 (20.9)	<.522
Hypertension	1257 (49)	453 (67.1)	<.001
Depression	389 (15.2)	93 (13.8)	.363
Autoimmune disease	115 (4.5)	5 (0.7)	<.001
Chronic lung disease	389 (15.2)	197 (29.2)	<.001
Thyroid disease	298 (11.6)	48 (7.1)	<.001
Heart failure	146 (5.7)	91 (13.5)	<.001
Renal failure	19 (0.7)	26 (3.8)	<.001
Drug abuse	239 (9.3)	124 (18.4	<.001

**Fig. 1:**
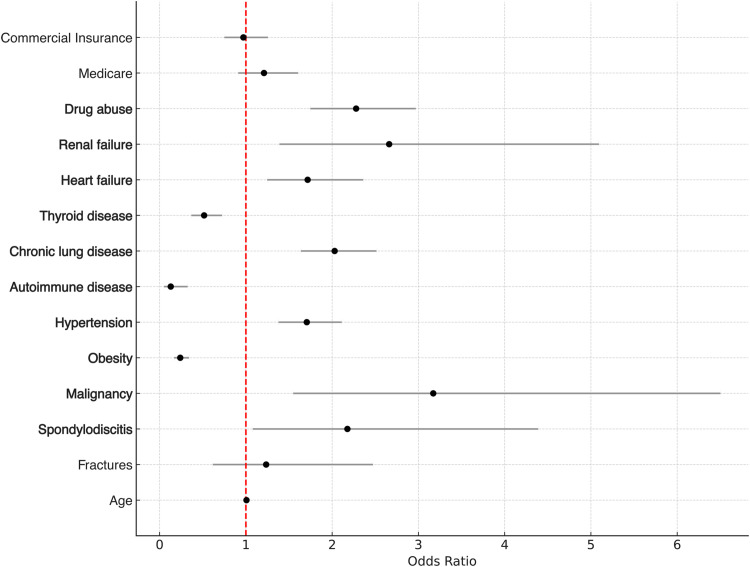
Forest plot of multivariate regression analysis. Forest plot visualizing the results of the multivariate regression analysis estimating the effects of various factors on 90-day readmission rates following lumbar corpectomy. Odds ratios (OR) and their 95% confidence intervals are presented for each variable. Significant predictors are marked in bold.

The multivariate regression analysis estimating the effects on readmission identified several significant risk factors (Table 3). The results of this analysis are visualized in Fig. 1. The highest odds predisposing for readmission were observed for malignancy of the spine (OR 3.172, p=.002). Spondylodiscitis was also a significant predictor for unplanned readmissions (OR 2.177, p=.030), whereas no significant association was found for fracture. Several medical comorbidities were also identified as significant risk factors, with renal failure, drug abuse and chronic lung disease having the highest odds. Conversely, obesity (OR 0.239, p<.001), autoimmune diseases (OR 0.129, p<.001), and thyroid diseases (OR 0.516, p<.001) were associated as significant factors against readmission.

**Table 3:** Multivariate regression analysis estimating the effects on readmission.

	Odds ratio	95% confidence intervals	p-value
Age	1.007	0.999–1.014	.069
Fractures	1.235	0.617–2.473	.551
Spondylodiscitis	2.177	1.08–4.389	.030
Malignancy	3.172	1.548–6.501	.002
Obesity	0.239	0.168–0.341	<.001
Hypertension	1.706	1.378–2.111	<.001
Autoimmune disease	0.129	0.051–0.325	<.001
Chronic lung disease	2.029	1.637–2.514	<.001
Thyroid disease	0.516	0.368–0.724	<.001
Heart failure	1.716	1.247–2.361	<.002
Renal failure	2.661	1.39–5.093	.003
Drug abuse	2.278	1.747–2.969	<.001
Medicare	1.210	0.912–1.605	<.186
Commercial Insurance	0.971	0.751–1.255	.823

Notably, no significant effects on the readmission rate were observed for age, Medicare or commercial insurance status.

## Discussion

Corpectomies are an essential part of the spine surgeon's armamentarium and usually reserved for more serious conditions requiring a more comprehensive decompression, deformity correction or anterior column resection and reconstruction. Arguably, lumbar corpectomies represent the most challenging anatomic region for reconstructive spine surgery due to their access and technical considerations. Although being subject to frequent research, current studies lack a general and comprehensive analysis of potential factors influencing readmission rates for lumbar corpectomies. Having realistic quantitative and qualitative parameters is foundational for clinicians and administrative professionals alike.

Previously published 90-day-all-cause readmission rates differ depending on procedure or underlying pathology. For elective lumbar spine surgery, 90-day-readmission rates from 2.5% up to 7.3% have been published, in contrast to a rate of 21.1% for adult degenerative pathologies and deformity surgery or 37% for metastatic diseases. We identified a 90-day all-cause readmission rate of 20.8% for lumbar corpectomy in patients with spondylodiscitis, fractures, or malignancy in the lumbar spine. Although high, this is within range of previously published rates [9,[12], [13], [14],^19^]. However, a rate of 20.8% underscores the severity of performing a corpectomy in contrast to a fusion, discectomy or laminectomy with a published readmission rate of 7.3% [13]. Excluding adult deformity and degenerate conditions in this study might overestimate the readmission rate as osteomyelitis and malignancy have recognized higher rates regardless of procedures performed [10].

Our analysis revealed malignancy and spondylodiscitis as independent predictors for readmission. Interestingly, no significant association was found for fractures, although being more frequent in the single admission group (66.3%) compared to the readmission group (46.1%, p<.001). Our findings suggest that underlying pathologies have dissimilar risk profiles, which may elucidate the previously discussed differences in readmission rates. This indicates that the condition might have a stronger influence on readmissions than the procedure performed.

The LOS during the initial admission did not differ significantly between the study groups but is notably 13.1 days longer compared to 4.49 days for primary elective spine surgery or adult deformity spine surgery [14,20]. This finding suggests that the severity of a surgical procedure may influence the LOS, as already shown in other surgical specialties. While previous studies have shown an extended LOS to be a risk factor for readmission and complications, we did not observe this association in our cohort [[21], [22], [23], [24]]. This may be due to our specific population studied, unmeasured confounding factors, or standardized postoperative care practices that mitigate the impact of LOS.

The Elixhauser indices differed significantly between the groups, indicating a higher comorbidity burden in the readmission group (p≤.001). Furthermore, our analysis identified numerous comorbidities that were more prevalent in the readmission group (Table 2). The effects of comorbidities on the readmission rate in spine surgery have been subject to many studies. Identified risk factors include diseases such as diabetes mellitus, hypertension, chronic lung diseases, drug abuse, obesity, depression and heart failure [13,14,19,20]. The findings of our study are consistent with the previously published literature on risk factors for the 90-day readmission rate.

Our analysis identified medical comorbidities, such as heart and renal failure as well as hypertension and drug abuse as independent risk factors (Table 3). Interestingly, one of the strongest predictors for readmission among the comorbidities was drug abuse (OR 2.278; p≤.001). This finding confirms the literature and underscores the challenges in managing dependency conditions, particularly in surgical patients. Substance abuse not only increases perioperative complications but contributes to inadequate follow-up care. Additionally, it is a recognized risk factor for spinal pathologies such as spondylodiscitis [25,26]. Renal failure (OR 2.661, p=.003), chronic lung disease (OR 2.029, p<.001) and heart failure (OR 1.716, p=.002) are also well-known predictors of adverse outcomes in spine surgery [10,13,14]. Noteworthy, obesity and thyroid diseases showed significant odds against the readmission group. A puzzling finding that we could not confirm was that obesity was not found to be adversely associated with readmissions in contrast to reported findings [14,19,27]. This discrepancy may be explainable by co-linearity among covariates in the statistical model, patient selection bias, or potential coding inaccuracies within the dataset.

Medicaid and commercial insurance showed no independent association with readmission. However, Medicare was significantly more frequent in the readmission group, whereas commercial insurance was more common in the single admission group. These findings are consistent with previous studies highlighting the influence of socioeconomic factors, comorbidity burden and availably of healthcare services on readmission rates and outcomes in spine surgery [28,29].

The limitations of this study mainly compose of the retrospective study design and utilization of large database analyses. These databases provide a broad dataset but may lack specific clinical data. Furthermore, same-day and outpatient procedures are also not captured. Coding inaccuracies or inconsistencies may affect the reliability and present a challenge, particularly in the context of degenerative and adult deformity conditions. For this reason, it was excluded. The definition of a corpectomy relied on multiple procedure codes rather than a single, standardized code. For the same reason, we did not report on surgical approaches or additional posterior procedures performed. Overall, this might have introduced potential bias, as unrelated conditions or procedures may have been included in the analysis, introducing a confounding error and influencing the statistical analysis. Using United States based data only may reduce generalizability across different populations or health care systems.

## Conclusions

A corpectomy of the lumbar spine is a widely used surgical procedure which may be used for a number of pathologies. Our study established an all-cause 90-day-readmission rate of 20.8% with a mean time to readmission of 38.34 (±22.3), which is within range of previously published data but also highlights the severity of this procedure. Our analysis revealed malignancy and spondylodiscitis as risk factors for readmission, whereas fractures were not. This underscores the risk profile of the underlying condition. In addition to that, several medical comorbidities showed a significant association as independent risk factors for readmission. These findings underscore the importance of preoperative assessment, management of comorbidities optimization where possible and considering socio-economic factors to reduce readmission rates and improve outcomes. However, the study's limitations may limit the generalizability of the findings.

All authors have given a written declaration of consent for publication of the data obtained in this study. I confirm that this work is original and has not been published elsewhere, nor is it currently under consideration for publication elsewhere.

## Availability of data and materials

The dataset is publicly available from the Healthcare Cost and Utilization Project (HCUP), Agency for Healthcare Research and Quality.

## Funding

This research received no specific grant from any funding agency in the public, commercial, or not-for-profit sectors. The authors have no relevant financial or nonfinancial interests to disclose.

## Ethics approval

This retrospective study utilizes data from the 2020 Nationwide Readmission Database (NRD), Healthcare Cost and Utilization Project (HCUP), Agency for Healthcare Research and Quality. Therefore, no approval from an institutional and national research committee was needed.

## Declaration of competing interests

One or more of the authors declare financial or professional relationships on ICMJE-TSJ disclosure forms.

## Appendix Supplementary materials

Image, application 1

Image, application 2

Image, application 3
