# Supplementary figures and images for: Spine surgery and readmission: Risk factors in lumbar corpectomy patients
Source: N Am Spine Soc J. 2025 Jan 20;21:100587. doi: 10.1016/j.xnsj.2025.100587 (PMC11848789; doi:10.1016/j.xnsj.2025.100587)

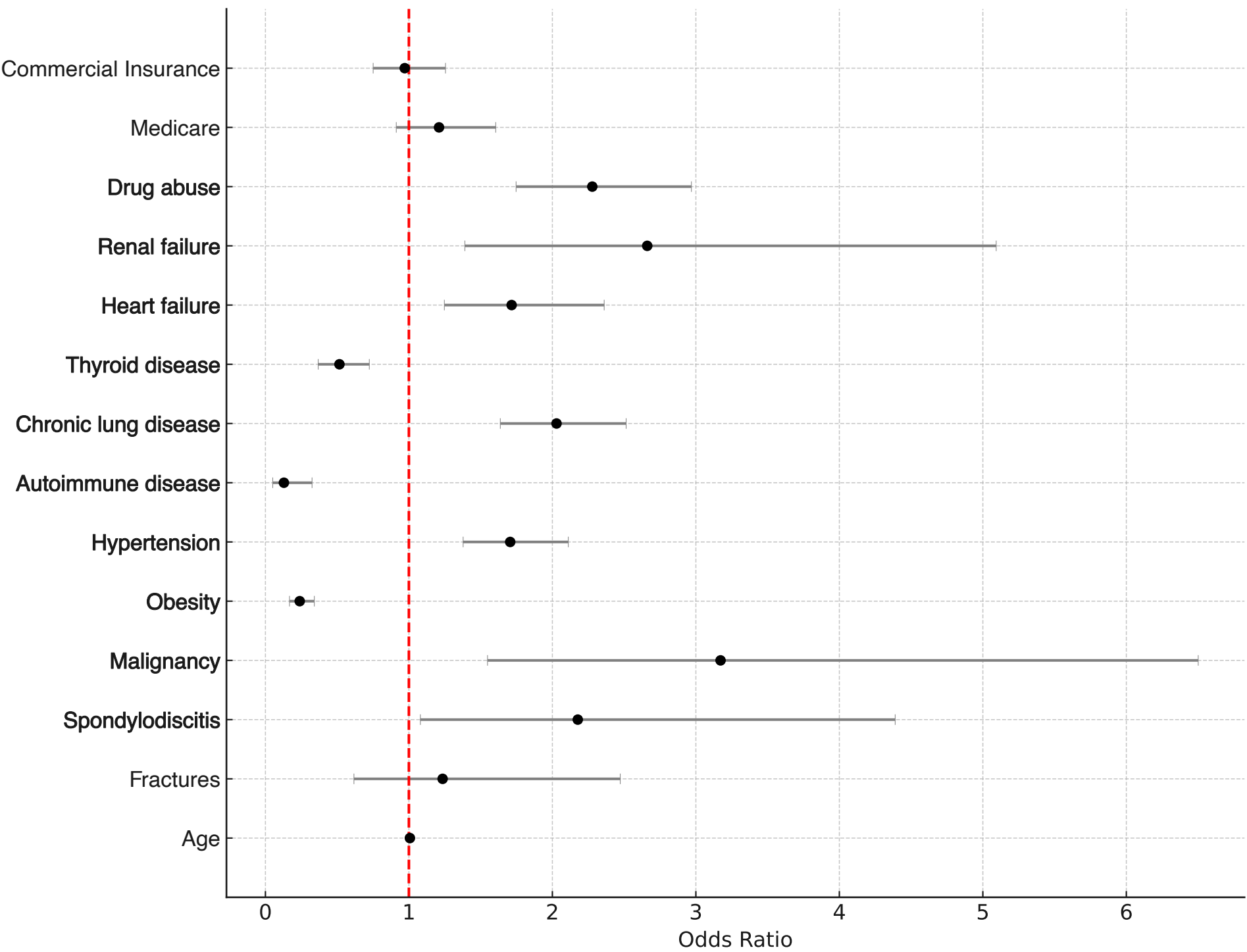

Supplement: Supplementary file 1 [file mmc1.pdf]
